# Towards Better Evaluation of Pneumococcal Vaccines

**DOI:** 10.1371/journal.pmed.0020053

**Published:** 2005-02-22

Pneumonia remains the leading cause of death worldwide in children. Several vaccines against pneumococcal pneumonia are at various stages of development, but the testing of their efficacy is hampered by the lack of noninvasive tests that are sensitive and specific for the disease. Diagnosis is usually based on chest radiographs, which are not very specific for pneumococcal disease.

In their quest for a more specific diagnostic test, Shabir Madhi and colleagues—who are conducting clinical trials on pneumococcal vaccines in children—examined whether serum concentrations of procalcitonin and C-reactive protein could improve the specificity of chest radiographs to diagnose pneumococcal pneumonia and thus be useful in the future evaluation of pneumococcal vaccines. Elevated levels of both proteins are associated with bacterial disease. They might therefore help to differentiate bacterial from nonbacterial causes of pneumonia, and thus allow to “enrich” the analyzed disease cases for those of pneumococcal origin, against which the vaccine is potentially active.

This study represents a first step, in which the researchers tested whether adding information about procalcitonin and C-reactive protein levels to data from a completed vaccine trial would affect the outcome regarding vaccine efficacy. When reanalyzing previous trial data under these conditions, the vaccine appeared more efficacious compared with placebo when either elevated procalcitonin or elevated C-reactive protein levels were taken into account. The efficacy estimate was greatest when cases of pneumonia that had elevated levels of both procalcitonin and C-reactive protein were compared against placebo.

These data suggest that elevated levels of C-reactive protein and procalcitonin, in conjunction with chest radiography, could improve the specificity of a diagnosis of pneumococcal pneumonia over that of chest radiography alone. This combined diagnostic test could be useful for further evaluation of pneumococcal vaccines. The hope is that among patients identified as having pneumonia by the combined test, a higher proportion would have pneumonia of pneumococcal origin. As a consequence, there would be less “background noise” caused by other forms of pneumonia, and this should make it easier to assess the efficacy of vaccine candidates. However, as the researchers point out, this analysis was not a primary objective of the present trial. This analysis can therefore serve only as a hypothesis-generating study, and as such the hypothesis must be tested in other study settings.

The study was sponsored by Wyeth, manufacturers of the pneumococcal vaccine used.

